# Iron Overload-Induced Ferroptosis Impairs Porcine Oocyte Maturation and Subsequent Embryonic Developmental Competence *in vitro*

**DOI:** 10.3389/fcell.2021.673291

**Published:** 2021-05-28

**Authors:** Weiyi Hu, Yan Zhang, Dali Wang, Tingting Yang, Jiajia Qi, Yonghong Zhang, Hao Jiang, Jiabao Zhang, Boxing Sun, Shuang Liang

**Affiliations:** ^1^Department of Animals Sciences, College of Animal Sciences, Jilin University, Changchun, China; ^2^Department of Animal Science, Chungbuk National University, Cheongju-si, South Korea

**Keywords:** iron overload, ferroptosis, porcine oocyte, oxidative stress, mitochondrial function

## Abstract

Accumulating evidence indicates that ferroptosis is an iron-dependent form of regulated cell death. This type of iron-dependent programmed cell death is different from traditional forms of regulated cell death, such as apoptosis and autophagy. However, the role of ferroptosis in porcine oocyte maturation and the associated mechanism remain unclear. In the present research, we investigated the effects of ferric ammonium citrate (FAC), a specific ferroptosis inducer, on porcine oocyte meiotic maturation and quality and subsequent embryonic developmental competence. FAC treatment caused obvious accumulation of intracellular ferrous ions in porcine oocytes. At the end of the *in vitro* maturation (IVM) period, there was a significant decrease in the polar body (PB) extrusion rate and an increase in the percentage of abnormal oocytes in the FAC treatment groups, indicating that iron overload-induced ferroptosis may suppress the meiotic process during porcine oocyte maturation. We also found that after FAC treatment, the subsequent two-cell rate, four-cell rate and blastocyst formation rate were significantly decreased in porcine parthenogenetic activation (PA) embryos, indicating that iron overload-induced ferroptosis decreased porcine oocyte quality. Further analysis revealed that FAC treatment not only enhanced intracellular reactive oxygen species (ROS) generation, decreased intracellular free thiol levels and induced mitochondrial dysfunction but also triggered autophagy in porcine oocytes. Taken together, these findings suggest that iron overload-induced ferroptosis impairs porcine oocyte meiosis and decreases porcine oocyte quality, possibly by increasing oxidative stress, inducing mitochondrial dysfunction and triggering autophagy.

## Introduction

With the development of livestock husbandry, an increasing number of assisted reproduction technologies, such as *in vitro* fertilization (IVF), somatic cell nuclear transfer (SCNT), and intracytoplasmic sperm injection (ICSI), have been widely used in the production of domestic animals. The implementation of these techniques needs to be accompanied by the use of high-quality *in vitro*- or *in vivo*-derived oocytes to be fully effective. Compared with *in vivo*-matured oocytes, *in vitro*-matured oocytes are easier to obtain. However, *in vitro*-matured oocytes are lower in quality and have a lower developmental potential than *in vivo*-matured oocytes. Oocyte *in vitro* maturation (IVM) is a complex process regulated by a large number of internal and external factors (Grupen, 2014). Any changes in this process lead to changes in oocyte quality, which affect the subsequent developmental capacity of preimplantation embryos (Koyama et al., 2014; Ahmed et al., 2017; Ferrer-Vaquer et al., 2019). Therefore, identifying the changes that occur in oocytes under stress conditions can help find potential solutions to reduce the corresponding negative effects.

Iron is a trace metal that is very important in mammalian physiological processes, such as DNA synthesis, energy generation, and oxygen transport, which rely on the existence of iron in variable and interconvertible oxidation states. However, dysregulation of iron homeostasis can lead to iron overload disorders, eventually resulting in excessive reactive oxygen species (ROS) generation and DNA damage and lipid peroxidation (Totsuka et al., 2019; Han et al., 2020; Tian et al., 2020). These events were defined as a form of programmed cell death called ferroptosis by Brent R. Stockwell’s team in 2012 (Dixon et al., 2012). Ferroptosis is a unique iron-dependent form of cell death (Xie et al., 2016; Tang et al., 2018) that is different from the traditional modes of cell death, such as necrosis (Pasparakis and Vandenabeele, 2015), autophagy (Glick et al., 2010), and apoptosis (Peña-Blanco and García-Sáez, 2018), in terms of cell morphology, biochemical characteristics and gene levels. Another characteristic of ferroptosis is the accumulation of ROS in cells (Sui et al., 2018). A large number of experiments have shown that ferroptosis occurs in neurodegenerative diseases (Abdalkader et al., 2018), infectious diseases (Matsushita et al., 2015), cancer (Chen et al., 2020), etc. Previous studies have found that under physiological and pathological conditions, a variety of hormonal (Belavgeni et al., 2019; Wang et al., 2019) and metabolic abnormalities (Stockwell et al., 2017; Wang et al., 2017) can trigger different types of cell death, including ferroptosis. Data from Zhang et al. (2020) showed that when the uterus and placenta of a female rat were dysfunctional, ferroptosis was triggered, and iron deposition occurred in the uterus. Furthermore, previous studies have also shown that the accumulation of iron and ferroptosis may occur in the early stage of follicular atresia (Zhang et al., 2018).

In the present research, a highly selective inducer of iron overload, ferric ammonium citrate (FAC), was used to establish an iron overload model in porcine oocytes. FAC, a trivalent iron salt, is absorbed *in vivo* by reducing trivalent iron to divalent ferrous iron (Cotticelli et al., 2019; Yao et al., 2021). It has been shown that FAC-induced intracellular iron overload causes ferroptosis (Fang et al., 2018). The aim of the present research was to determine whether iron overload-induced ferroptosis during IVM impairs meiotic maturation and developmental competence of porcine oocytes.

## Materials and Methods

All chemicals used in this research were obtained from Sigma-Aldrich (St. Louis, MO, United States) unless otherwise noted.

### Oocyte Collection and IVM

Porcine ovaries were obtained from a local slaughterhouse and transported to the laboratory in sterile 0.9% saline at 30–35°C. Cumulus-oocyte complexes (COCs) were obtained by aspirating 3∼8 mm antral follicles with a syringe. COCs with at least three or more layers of uniformly distributed cumulus cells were collected using Tyrode’s lactate-hydroxyethylpiperazine ethane sulfonic acid (HEPES) medium supplemented with 0.1% polyvinyl alcohol (PVA, w/v) and 0.05 g/L gentamycin under a stereomicroscope (S22-LGB, Nikon). The IVM medium consisted of tissue culture medium 199 (TCM-199, Invitrogen, Carlsbad, CA, United States) supplemented with 10% (v/v) porcine follicular fluid, 10 IU/mL follicle stimulating hormone (Ningbo No. 2 Hormone Factory, China), 10 IU/mL luteinizing hormone (Ningbo No. 2 Hormone Factory, China), 0.91 mM Na pyruvate, 10 ng/mL EGF, and 75 mg/mL kanamycin. The IVM medium was completely covered with mineral oil and cultured in an incubator containing 5% CO_2_ at 100% humidity at 38.5°C for 42 h.

For FAC treatment, FAC powder was dissolved in IVM medium at a concentration of 20 μM in the dark, and then 20 μM FAC solution was diluted in IVM medium to obtain 5 μM and 10 μM FAC solutions.

### Parthenogenetic Activation (PA) and *In vitro* Culture (IVC)

Porcine oocyte PA was induced according to our previously described procedures (Qi et al., 2020). Briefly, cumulus cells were removed from COCs with cumulus cells expanded by 0.1% hyaluronidase at the end of the IVM period. Polar body (PB) extrusion of oocytes was examined under a stereomicroscope. The denuded oocytes were then subjected to electrical activation [300 mM mannitol containing 0.1 mM CaCl_2_, 0.05 mM MgSO_4_, 0.01% PVA (w/v), and 0.5 mM HEPES] at 110 V and 60 μs twice. After that, these oocytes were transferred to IVC medium [bicarbonate-buffered porcine zygote medium (PZM)-5 (Suzuki et al., 2007) comprising 4 mg/mL BSA] supplemented with 7.5 μg/mL cytochalasin B and cultured for 3 h to suppress extrusion of the pseudo-second PB. Next, the oocytes were thoroughly washed and cultured in IVC medium in four-well plates covered with mineral oil and cultured for 6.5 days at 38.5°C under 100% humidity and an atmosphere of 5% CO_2_ without changing the medium. Two-cell, four-cell and blastocyst formation rates were analyzed under a stereomicroscope at 24 h, 48 h, and 6.5 days. The two-cell, four-cell, and blastocyst formation rates were calculated by the number of examined embryos to the total embryos in each group.

### Ferrous Ion Staining

Intracellular Fe^2+^ levels were examined at the end of the IVM period. The oocytes in each group were thoroughly washed in prewarmed PBS-PVA medium and assessed using the fluorescent probe Ferro Orange (Dojindo, F374) for 30 min. Images of the fluorescence signals were captured as TIFF files using a digital camera connected to a fluorescence microscope. The same procedures were followed for all groups of oocytes, including incubation, rinsing, mounting, and imaging. The fluorescence signal intensities of the oocytes in each group were analyzed via National Institutes of Health (NIH) ImageJ software (NIH, Bethesda, MD, United States).

### Intracellular ROS Levels, Free Thiol Levels

Intracellular ROS levels and free thiol levels in oocytes were measured with an ROS detection kit (Thermo Fisher Scientific, C400) and free thiol level detection kit (Thermo Fisher Scientific, C12881). To determine intracellular ROS levels, oocytes were incubated for 15 min in PBS-PVA medium containing 10 μM 2′,7′-dichlorodihydrofluorescein diacetate. To determine intracellular free thiol levels, oocytes were incubated for 30 min in PBS-PVA medium containing 10 μM CMF2HC. Fluorescent signals were captured as a TIFF file using a digital camera connected to a fluorescence microscope. The same procedures were followed for all groups of oocytes, including incubation, rinsing, mounting, and imaging. The fluorescence signal intensities of the oocytes in each group were analyzed via NIH ImageJ software.

### Mitochondrial Membrane Potential (MitoMP) Assessment

Mitochondrial membrane potential in oocytes was measured with a JC-1 MitoMP detection kit (Dojindo, MT09). Briefly, oocytes were incubated in PBS-PVA containing 2 μM JC-1 for 30 min. The MitoMP was calculated as a ratio of red florescence (J-aggregates; corresponding to activated mitochondria) to green fluorescence (J-monomers; corresponding to less active mitochondria). Images of the fluorescence signals were captured as TIFF files using a digital camera connected to a fluorescence microscope. The same procedures were followed for all groups of oocytes, including incubation, rinsing, mounting, and imaging. The fluorescence signal intensities of the oocytes in each group were analyzed via NIH ImageJ software.

### Intracellular ATP Level Measurement

Intracellular ATP levels were measured using an ATP Detection Kit (Beyotime, S0027). Briefly, porcine oocytes from each group were collected and lysed with 200 μL of lysis buffer at the end of the IVM period. Next, the cell lysates were centrifuged at 12000 rpm at 4°C for 5 min, and the supernatant was taken for subsequent analysis. Then, 100 μL of ATP working solution and 20 μL of supernatant were added to 96-well opaque plates, which were analyzed with a luminometer (Tecan, Infinite M200 Pro).

### Western Blotting Analysis

For Western blotting, 100 oocytes from each group were collected and fully lysed at 95°C in lysis buffer comprising 10% Tris–HCl, 40% DDH2O, 50% glycerol, 0.5 mM Tris–HCl, β-mercaptoethanol, and bromophenol blue. The protein samples were then loaded in a 10% polyacrylamide gel containing 0.1% SDS, and the separated proteins were transferred to polyvinylidene fluoride (PVDF) membranes (Millipore). The PVDF membranes were blocked in 5% BSA at room temperature for 2 h and then incubated with primary antibodies against GAPDH (CST, #2118S), β-tubulin (Proteintech, 10094-1-AP), caspase-3 (Wanleibio, WL02117), Bcl-2 (Wanleibio, WL01556), Bax (Wanleibio, WL01637), GPX4 (BOSTER, BM5231), and LC-3 (CST, #11972S). After being washed with 1x TBST for 5 min each four times, the membranes were incubated at room temperature for 1 h with horseradish peroxidase-conjugated goat anti-rabbit IgG (Bioworld Technology, Inc., Louis Park, MN, United States, BS13278). The blots were visualized and analyzed by using a Tanon 5200 Image Analyzer (Tanon, Shanghai, China) and NIH ImageJ software, respectively.

### Statistical Analysis

SPSS software version 11.0 (IBM, United States) was used to analyze all the data collected. Comparisons of data among groups were performed using one-way ANOVA or Student’s *t*-test. The results are presented as the mean ± standard error of mean (SEM) of the mean. Significant differences are indicated by different letters (*p* < 0.05).

## Results

### FAC Treatment Results in Intracellular Fe^2+^ Accumulation and Deterioration of Porcine Oocyte Quality

**FIGURE 1:**
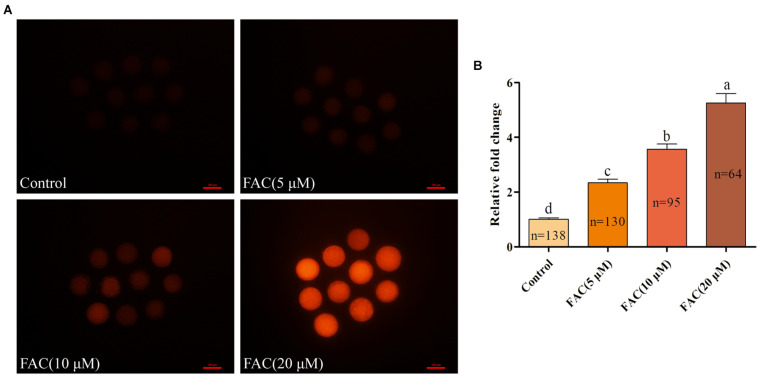
Effects of FAC on intracellular Fe^2+^ accumulation in porcine oocytes during IVM. **(A)** Representative images of the fluorescent probe FerroOrange showing intracellular Fe^2+^ levels in porcine oocytes. Scale bar = 100 μm. **(B)** Quantification of the relative intracellular Fe^2+^ levels in porcine oocytes from the different FAC treatment groups. The number of oocytes examined from each experimental group is indicated by the bars. Statistically significant differences are represented by different letters (*p* < 0.05).

**FIGURE 2:**
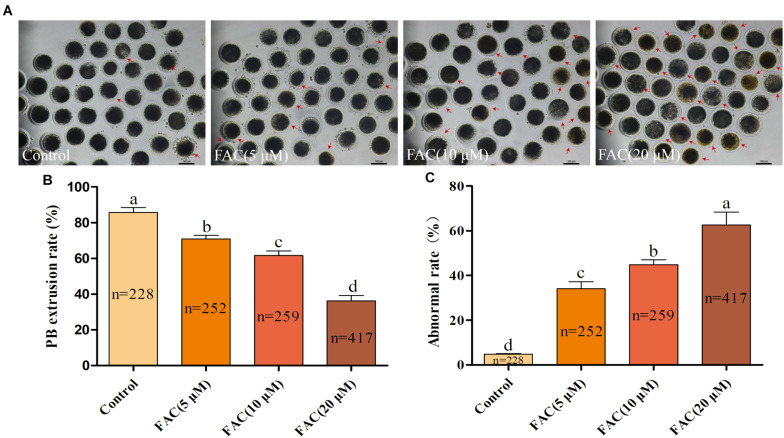
FAC treatment impairs the porcine oocyte maturation process. **(A)** Representative images of porcine oocytes treated with different concentrations of FAC at the end of the IVM period are shown. Porcine oocytes with morphological abnormalities as examined by optical microscopy are indicated by arrows. Scale bar = 100 μm. **(B)** Oocyte PB extrusion rate in each experimental group. **(C)** Percentage of abnormal oocytes in each experimental group. The number of oocytes examined from each experimental group is indicated by the bars. Statistically significant differences are represented by different letters (*p* < 0.05).

To investigate the potential involvement of ferroptosis in oocyte quality during IVM, porcine oocytes were treated with increasing concentrations of FAC (5 μM, 10 μM, and 20 μM), and intracellular Fe^2+^ levels, the rate of PB extrusion and the percentage of abnormal oocytes (Supplementary Figure 1) were analyzed. Analysis with the fluorescence probe FerroOrange revealed that the relative intracellular Fe^2+^ levels in oocytes increased in a concentration-dependent manner (Figures 1A,B). Further analysis revealed that FAC treatment decreased the rate of maturation (85.74 ± 2.69%, 70.86 ± 2.11%, 61.58 ± 2.66%, 36.27 ± 3.02%; *p* < *<*0.05) and increased the percentage of abnormal oocytes (4.80 ± 0.32%, 34.05 ± 3.19%, 44.84 ± 2.20%, 62.62 ± 5.75%; *p* < *<*0.05) in a dose-dependent manner (Figures 2A–C). In addition, FAC treatment impaired cumulus cell expansion capacity in porcine oocytes (Supplementary Figure 2). Western blotting analysis showed that the expression of the key ferroptosis factor GPX4 was upregulated, but there was no statistical significance in the expression of the apoptosis-related factors cleaved-caspase-3, BCL-2 and BAX in oocytes treated with FAC compared with oocytes in the control group (Supplementary Figures 3, 4). These results suggest that iron overload-induced ferroptosis has a direct negative effect on the porcine oocyte maturation process. According to our pre-experiment, 10 μM FAC was used for all subsequent experiments.

### Effects of FAC Treatment During IVM on Subsequent *In vitro* Embryo Development After PA

**FIGURE 3:**
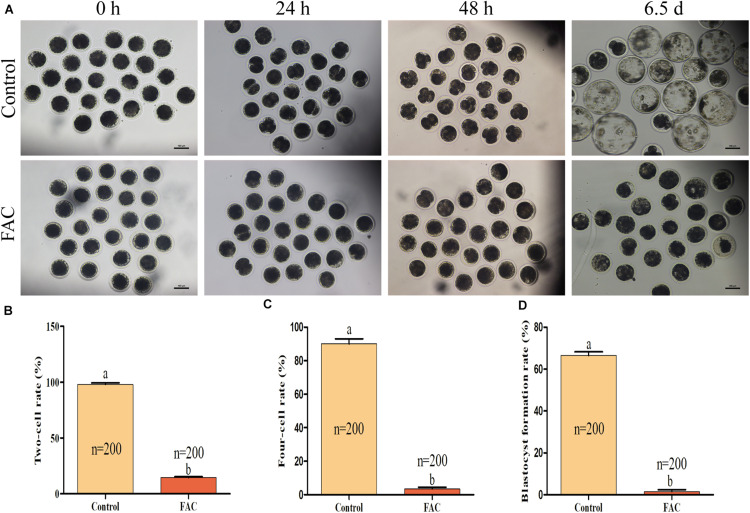
Developmental competence of porcine oocytes after FAC treatment. **(A)** Development of PA embryos from the control and FAC treatment groups at different time points. Scale bar = 100 μm. **(B)** Two-cell rate of PA embryos from the control and FAC treatment groups. **(C)** Four-cell rate of PA embryos from the control and FAC treatment groups. **(D)** Blastocyst formation rate of PA embryos from the control and FAC treatment groups. The number of embryos examined from each experimental group is indicated by the bars. Statistically significant differences are represented by different letters (*p* < 0.05).

Since the quality of an oocyte directly affects its developmental potential, we next assessed whether FAC treatment during the IVM period decreased the developmental competence of porcine PA embryos. The results showed that FAC treatment had a negative effect on porcine embryo developmental competence (Figure 3A). The two-cell rates (Figure 3B; 98.00 ± 1.41% vs. 14.50 ± 0.96% at 24 h; *p* < 0.05), four-cell rates (Figure 3C; 90.00 ± 3.16% vs. 3.50 ± 0.96% at 48 h; *p* < 0.05), and blastocyst formation rates (Figure 3D; 66.50 ± 1.71% vs. 1.50 ± 0.96% on day 6.5; *p* < 0.05) of the PA embryos generated from mature oocytes from the FAC-treated group were significantly lower than those of PA embryos generated from mature oocytes from the control group.

### Effects of FAC Treatment During IVM on the Oxidative Resistance of Porcine Oocytes

**FIGURE 4:**
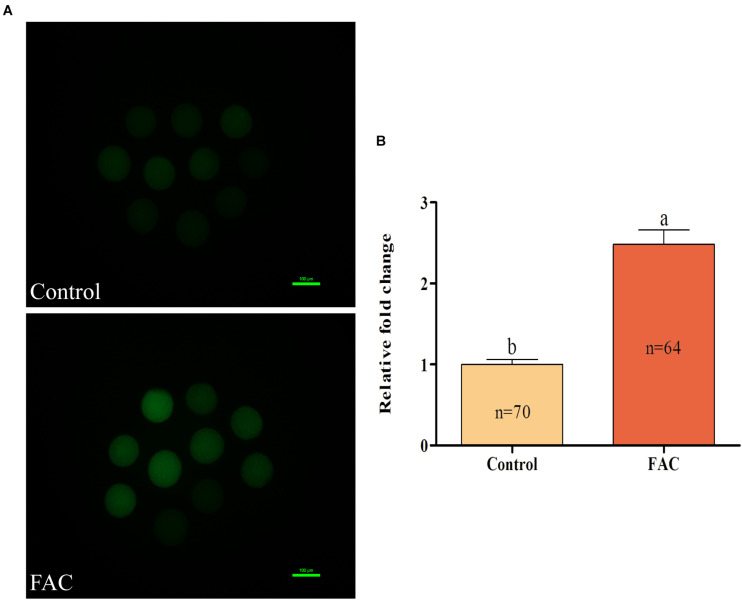
Effects of FAC treatment on intracellular ROS generation in porcine oocytes during IVM. **(A)** Representative fluorescence images showing intracellular ROS levels in porcine oocytes. Scale bar = 100 μm. **(B)** Quantification of relative intracellular ROS levels in porcine oocytes from the control and FAC treatment groups. The number of oocytes examined from each experimental group is indicated by the bars. Statistically significant differences are represented by different letters (*p* < 0.05).

**FIGURE 5:**
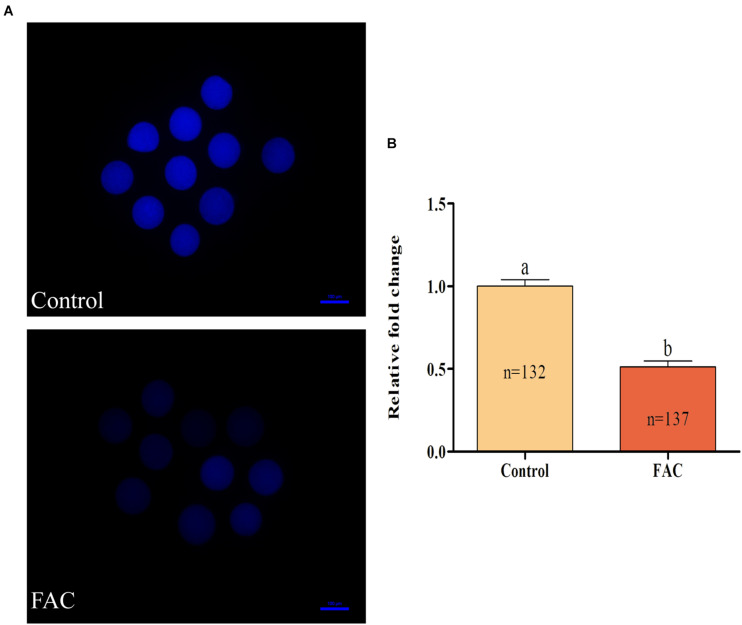
Effects of FAC treatment on intracellular free thiol levels in porcine oocytes during IVM. **(A)** Representative fluorescence images showing intracellular free thiol levels in porcine oocytes. Scale bar = 100 μm. **(B)** Quantification of relative intracellular free thiol levels in porcine oocytes from the control and FAC treatment groups. The number of oocytes examined from each experimental group is indicated by the bars. Statistically significant differences are represented by different letters (*p* < 0.05).

To analyze the mechanism through which FAC-induced ferroptosis affects porcine oocyte maturation, intracellular ROS and free thiol levels in FAC-treated oocytes were measured. Intracellular ROS levels were measured by assessing DCFH fluorescence (Figure 4A). Quantitative analysis showed that the relative intracellular ROS levels in porcine oocytes were significantly increased in the FAC treatment group compared with the control group (Figure 4B; *p* < 0.05). Next, the intracellular free thiol levels in porcine oocytes were measured (Figure 5A). As shown in Figure 5B, quantitative analysis showed that the relative intracellular free thiol levels were significantly lower in the FAC treatment group than in the control group (*p* < 0.05), suggesting that iron overload-induced ferroptosis can lead to oxidative stress and decrease the oxidative resistance of porcine oocytes.

### Effects of FAC Treatment During IVM on Mitochondrial Function in Porcine Oocytes

**FIGURE 6:**
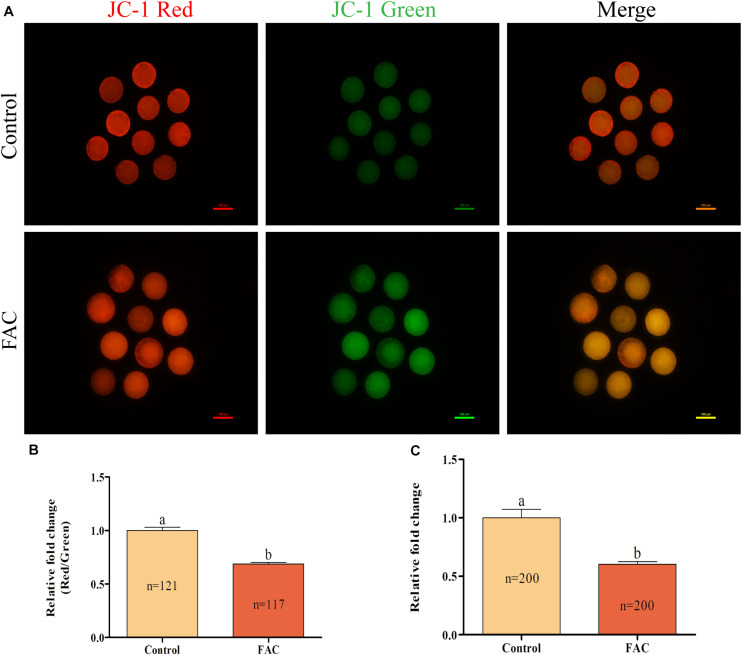
Effects of FAC treatment on mitochondrial function in porcine oocytes during IVM. **(A)** Representative fluorescence images of JC-1-stained porcine oocytes. Scale bar = 100 μm. **(B)** Quantification of the relative JC-1 fluorescence intensity in porcine oocytes from the control and FAC treatment groups. **(C)** Quantification of relative intracellular ATP levels in porcine oocytes from the control and FAC treatment groups. The number of oocytes examined from each experimental group is indicated by the bars. Statistically significant differences are represented by different letters (*p* < 0.05).

As the source of energy for cells, mitochondria play a vital role in the oocyte maturation process. Therefore, the intracellular MitoMP and ATP levels in porcine oocytes were analyzed. The intracellular MitoMP of porcine oocytes was evaluated using JC-1 fluorescent dye (Figure 6A). Quantitative analysis showed that the relative intracellular MitoMP of porcine oocytes was decreased in the FAC treatment group compared with the control group (Figure 6B; *p* < 0.05). Further analysis showed that the relative intracellular ATP levels in porcine oocytes were significantly lower in the FAC treatment group than in the control group (Figure 6C; *p* < 0.05). These results indicate that iron overload-induced ferroptosis can impair mitochondrial function in porcine oocytes.

### Effects of FAC Treatment During IVM on Autophagy in Porcine Oocytes

**FIGURE 7:**
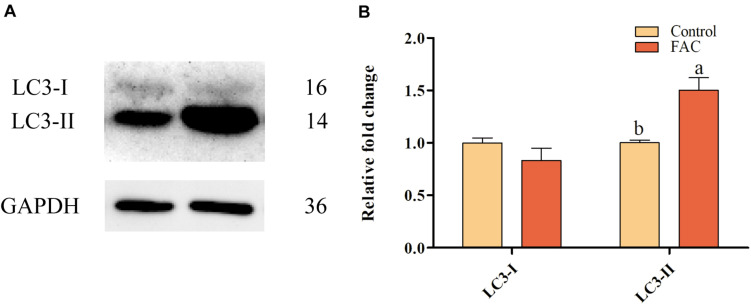
Effects of FAC treatment on autophagy in porcine oocytes during IVM. **(A)** Western blotting analysis of LC3 expression in porcine oocytes. **(B)** Quantitative LC3-I and LC3-II levels in porcine oocytes. Statistically significant differences are represented by different letters (*p* < 0.05).

To evaluate whether FAC-induced ferroptosis can induce autophagy in porcine oocytes, the protein expression of LC3, which is associated with autophagy, in porcine oocytes was analyzed after FAC treatment. Western blotting analysis showed that LC3-II protein expression was upregulated in oocytes treated with FAC compared with oocytes in the control group (Figure 7). This result indicates that iron overload is related to the induction of autophagy in porcine oocytes.

## Discussion

The present research suggested that iron overload disorders induced by FAC decreased porcine oocyte quality by increasing intracellular ROS generation, decreasing intracellular free thiol levels, and inducing mitochondrial dysfunction during IVM. Importantly, subsequent embryonic developmental potential was markedly decreased following iron overload during IVM of porcine oocytes. These results suggest that dysregulation of iron homeostasis decreases porcine oocyte quality and subsequent embryonic developmental competence.

Iron overload induces ferroptosis characterized by phospholipid peroxidation of plasma membranes caused by ROS generated during iron-mediated Fenton reactions (Dixon et al., 2012). A previous study showed that iron overload induces ferroptosis in cells and a loss of antioxidant defense (Chen et al., 2021). Porcine oocytes have relatively higher intracellular lipid levels than oocytes of other species, making them highly sensitive to ROS-induced impairments (Gajda, 2009). A previous study suggested that excessive intracellular ROS accumulation can induce cell cycle arrest and apoptosis in oocytes (Tripathi et al., 2009; Tiwari et al., 2017). It was found that oxidative stress can lead to a decrease in oocyte quality and reduce subsequent embryonic developmental competence (Yu et al., 2019; Zhou et al., 2019). In the present study, we found that FAC-induced iron overload led to intracellular ROS generation in porcine oocytes. To further evaluate the underlying process and mechanism through which FAC-induced iron overload decreases the quality and developmental potential of porcine oocytes, we examined intracellular free thiol levels. The levels of intracellular free thiols are regarded as important indicators of cytoplasmic maturation of oocytes at the end of the IVM period (Liang et al., 2018; Zhang et al., 2019). Several studies have shown that oocytes with higher intracellular ROS levels have lower intracellular free thiol levels and insufficient embryonic developmental potential (Nabenishi et al., 2012; Liang et al., 2017b; Li and Zhao, 2019). In the present study, FAC-induced iron overload during IVM decreased intracellular free thiol levels in the cytoplasm. These results are consistent with our hypothesis that FAC-induced iron overload decreases porcine oocyte quality by consuming intracellular free thiols and inducing the accumulation of intracellular ROS.

Mitochondria are a site of energy metabolism and are involved in cell apoptosis and death. Several studies have shown that mitochondrial function influences oocyte developmental potential and is associated with subsequent embryonic development, such as that of PA, IVF, and SCNT embryos (Liang et al., 2018; An et al., 2019; Hu et al., 2020; Nie et al., 2020). Recent research has suggested that ferroptosis can lead to mitochondrial dysfunction, including loss of the MitoMP, enhanced mitochondrial fragmentation, and reduced mitochondrial respiration, in neuronal HT22 cells and mouse embryonic fibroblasts (Jelinek et al., 2018). In addition, *in vivo* studies have suggested that excessive iron accumulation induces ferroptosis, not only exacerbating mitochondrial dysfunction but also increasing intracellular ROS and malondialdehyde levels (Kumfu et al., 2016, 2018; Wongjaikam et al., 2016, 2017; Khamseekaew et al., 2017; Sumneang et al., 2020). Abdalkader et al. (2018) also found that the characteristics of many neurodegenerative diseases are similar to those of ferroptosis-associated conditions, such as iron accumulation disorders and mitochondrial dysfunction. The MitoMP is commonly used as an indicator of mitochondrial function in oocytes (Liang et al., 2018) and is the driving force behind intracellular ATP synthesis (Dimroth et al., 2000). There is increasing evidence that oocytes with a higher MitoMP have better developmental potential (Liang et al., 2017a; An et al., 2019; Nie et al., 2020; Niu et al., 2020). Previous studies have suggested that iron overload could induce apoptosis through mitochondrial dysfunction, which increased mitochondrial oxidative stress and activated the caspase-dependent apoptotic pathway (Khamseekaew et al., 2017; Kumfu et al., 2018). Therefore, we analyzed intracellular MitoMP and ATP production in porcine oocytes after FAC treatment. Mitochondrial functional assays revealed that intracellular MitoMP and ATP production exhibited significant decreasing trends. These changes may account for the decrease in the quality of porcine oocytes after FAC-induced iron overload as well as the reduction in oocyte developmental potential. A similar study of mouse spermatozoa revealed that iron overload significantly decreases motility, viability, MitoMP, and GPX activity and increases the generation of ROS (Mojica-Villegas et al., 2014). Further studies, including studies on mitochondrial dysfunction and transmission electron microscopy results of mitochondria, are needed to further investigate the mechanism by which iron overload decreases the developmental competence of oocytes in pigs.

Autophagy is an intracellular process of self-degradation that occurs in abnormal physiological processes. LC3 is an autophagosome-labeling protein. LC3I exists in two forms: LC3I is lipidated and ubiquitylated into LC3II, which is ultimately targeted to the autophagosome or its precursor (Kabeya et al., 2000). It has been suggested that the modification of LC3I to LC3II is a sign of autophagy (Kabeya et al., 2004). In the present research, FAC-induced iron overload upregulated the expression of LC3II in porcine oocytes. This result was consistent with previous studies showing that iron overload-induced ferroptosis triggers autophagy in L6 skeletal muscle cells (Jahng et al., 2019) and murine preosteoblast cells (Cen et al., 2018). Thus, iron overload-induced ferroptosis might trigger autophagy to affect porcine oocyte meiotic maturation and block further development.

## Conclusion

Taken together, the present research demonstrated that iron overload-induced ferroptosis might decrease porcine oocyte quality by inducing intracellular ROS generation and decreasing intracellular free thiol levels and mitochondrial dysfunction. These findings provide novel insights into the mechanisms underlying iron overload-induced ferroptosis in oocytes. In the future, *in vivo* experiments should be carried out to confirm the effect of iron overload-induced ferroptosis on porcine oocyte maturation and reduce the limitations of *in vitro*-matured oocyte tests.

## Data Availability Statement

The original contributions presented in the study are included in the article/Supplementary Material, further inquiries can be directed to the corresponding authors.

## Ethics Statement

The present research followed the Care and Use of Laboratory Animals prepared by the Institutional Animal Care and Use Committee of Jilin University, China.

## Author Contributions

WH, YaZ, BS, and SL participated in the research design and wrote the article. WH, YaZ, DW, TY, and JQ participated in the experiment and data analysis. WH, YoZ, SL, HJ, and JZ participated in revising the article. All authors approved the submitted version.

## Conflict of Interest

The authors declare that the research was conducted in the absence of any commercial or financial relationships that could be construed as a potential conflict of interest.
