# Supplementary figures and images for: Iron Overload-Induced Ferroptosis Impairs Porcine Oocyte Maturation and Subsequent Embryonic Developmental Competence in vitro
Source: Front Cell Dev Biol. 2021 May 28;9:673291. doi: 10.3389/fcell.2021.673291 (PMC8194094; doi:10.3389/fcell.2021.673291)

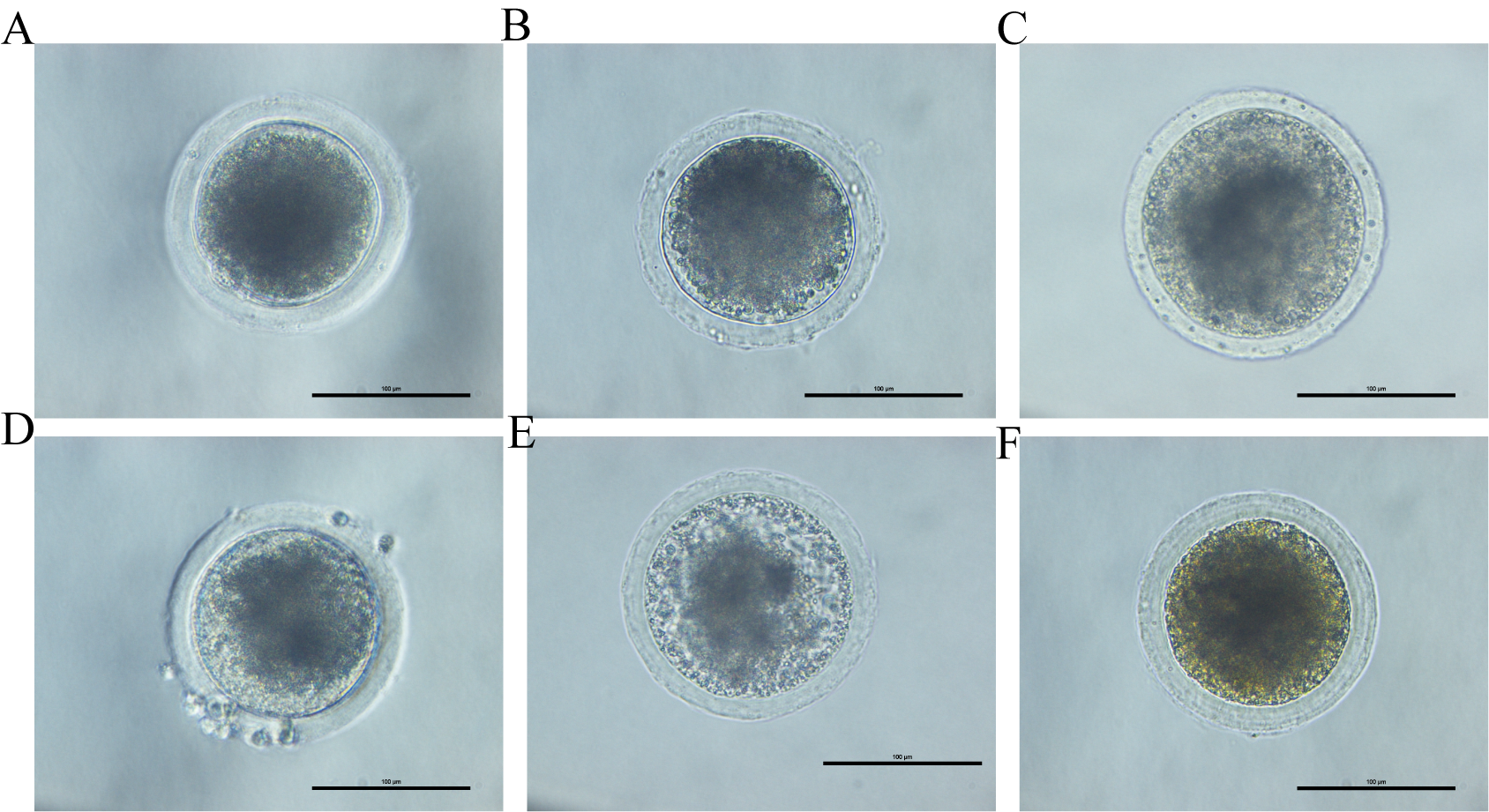

Supplement: Supplementary file 1 [file Image_1.TIF]

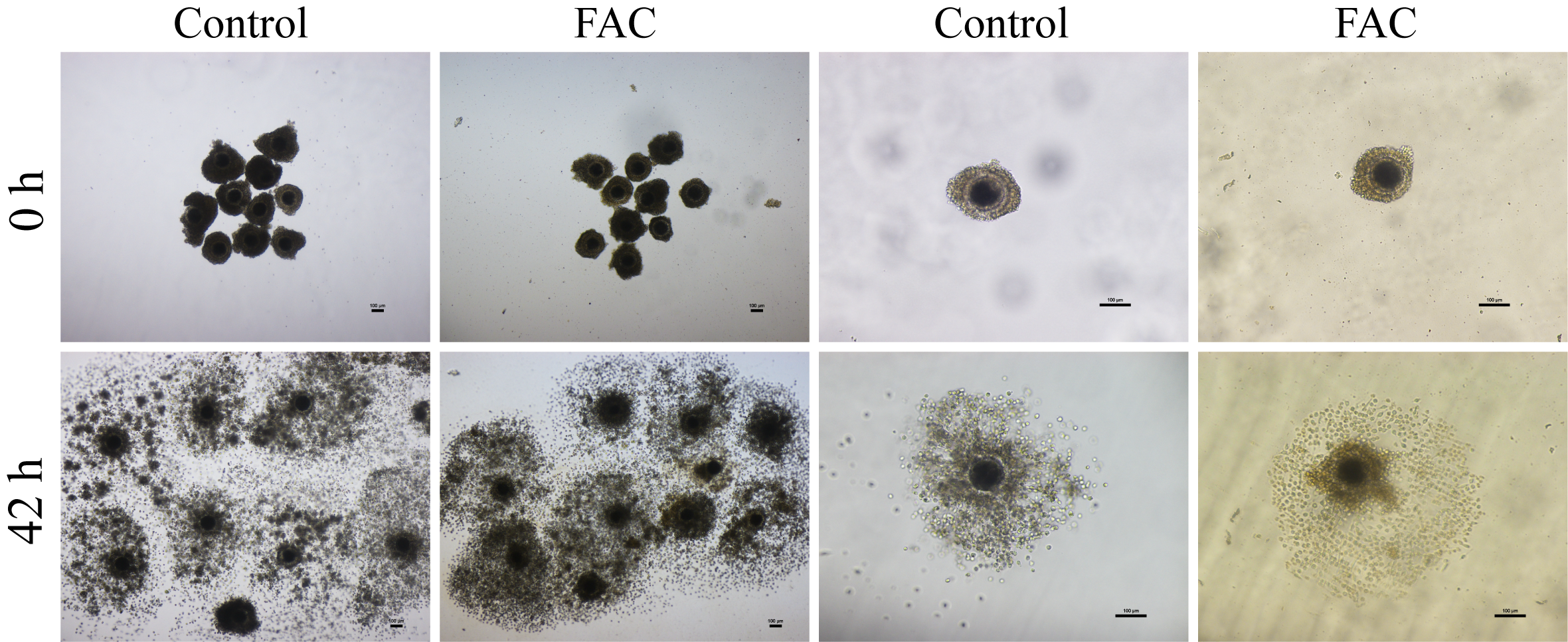

Supplement: Supplementary file 2 [file Image_2.TIF]

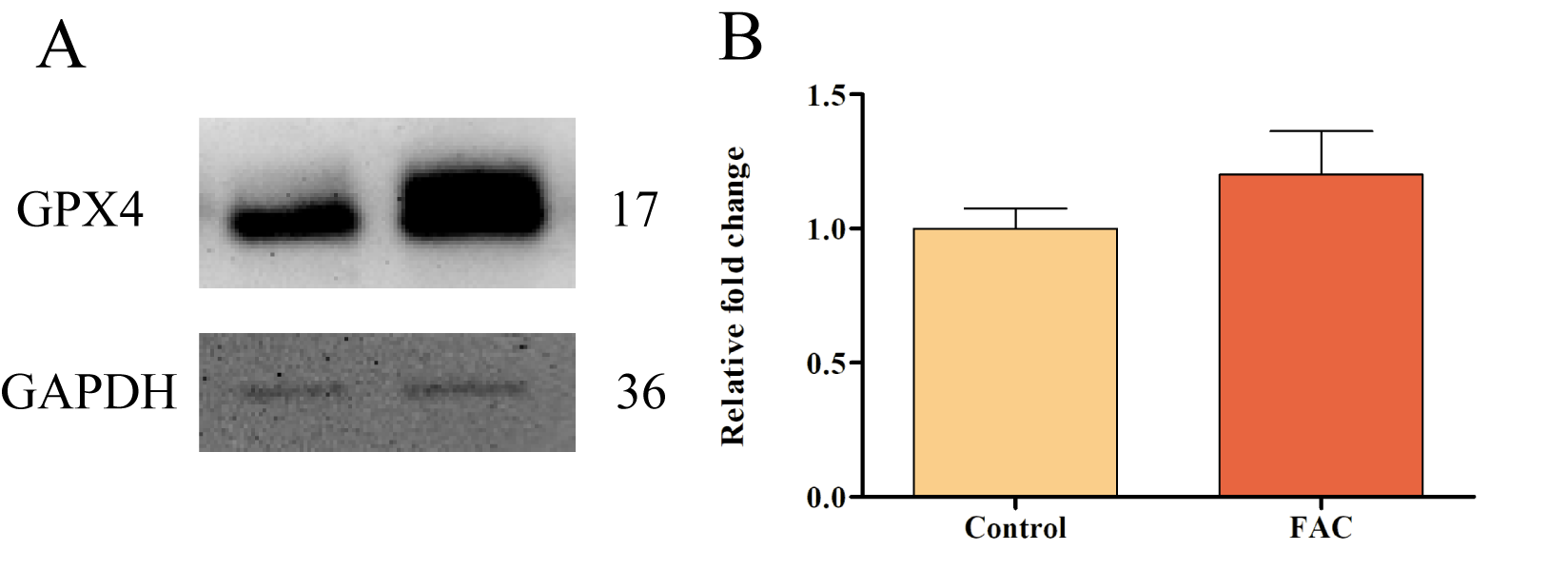

Supplement: Supplementary file 3 [file Image_3.TIF]

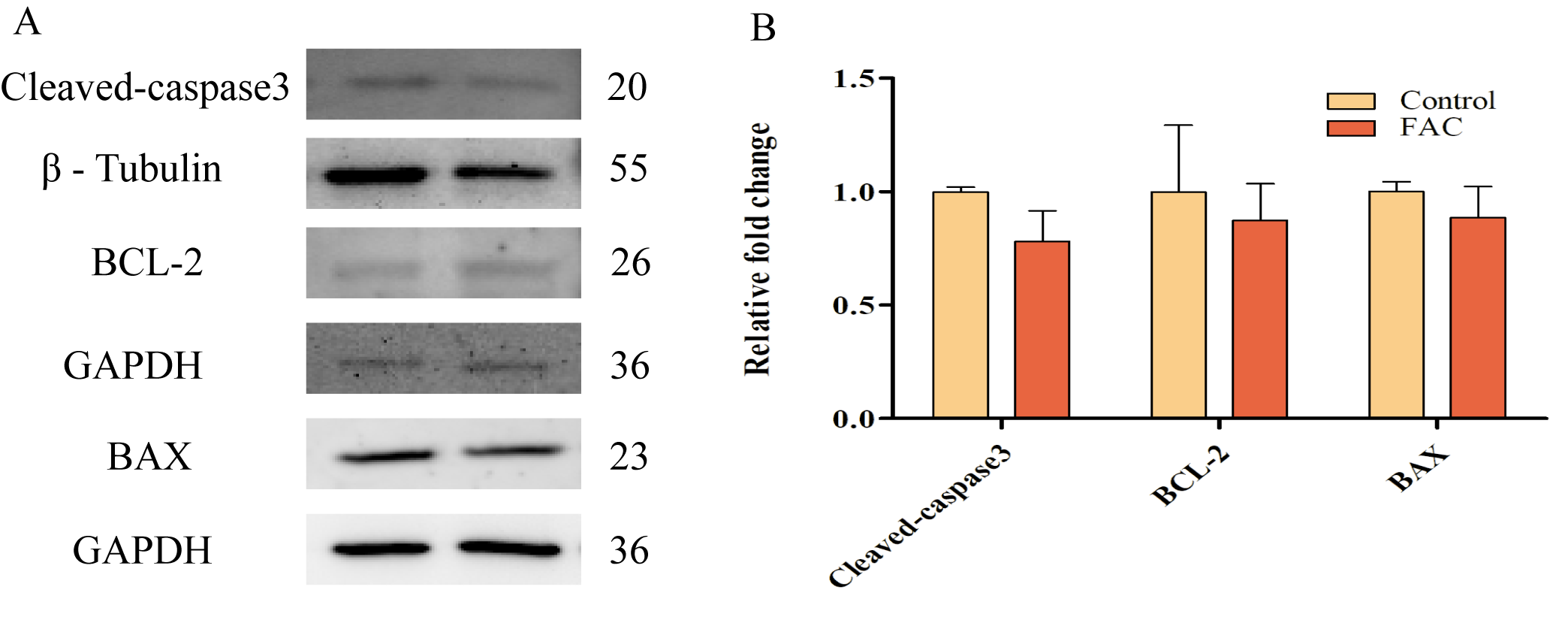

Supplement: Supplementary file 4 [file Image_4.TIF]
